# Author Correction: An extensive disulfide bond network prevents tail contraction in *Agrobacterium tumefaciens* phage Milano

**DOI:** 10.1038/s41467-025-62720-y

**Published:** 2025-08-07

**Authors:** Ravi R. Sonani, Lee K. Palmer, Nathaniel C. Esteves, Abigail A. Horton, Amanda L. Sebastian, Rebecca J. Kelly, Fengbin Wang, Mark A. B. Kreutzberger, William K. Russell, Petr G. Leiman, Birgit E. Scharf, Edward H. Egelman

**Affiliations:** 1 Department of Biochemistry and Molecular Genetics, University of Virginia School of Medicine, Charlottesville, VA 22903 USA; 2 Mass Spectrometry Facility, University of Texas Medical Branch, Galveston, TX 77555 USA; 3 Department of Biological Sciences, Virginia Tech, Blacksburg, VA 24061 USA; 4 Department of Biochemistry and Molecular Biology, University of Texas Medical Branch, Galveston, TX 77555 USA; 5 Present Address: Department of Biochemistry and Molecular Genetics, University of Alabama at Birmingham, Birmingham, AL 35233 USA

**Keywords:** Supramolecular assembly, Cryoelectron microscopy, Bacteriophages

Correction to: *Nature Communications* 10.1038/s41467-024-44959-z, published online 26 January 2024

The original version of the Supplementary Information associated with this Article contained an error in Figure S5. The distance values of the contracted and extended phage tail power spectrum have been incorrectly labeled.

The correct version of Figure S5:
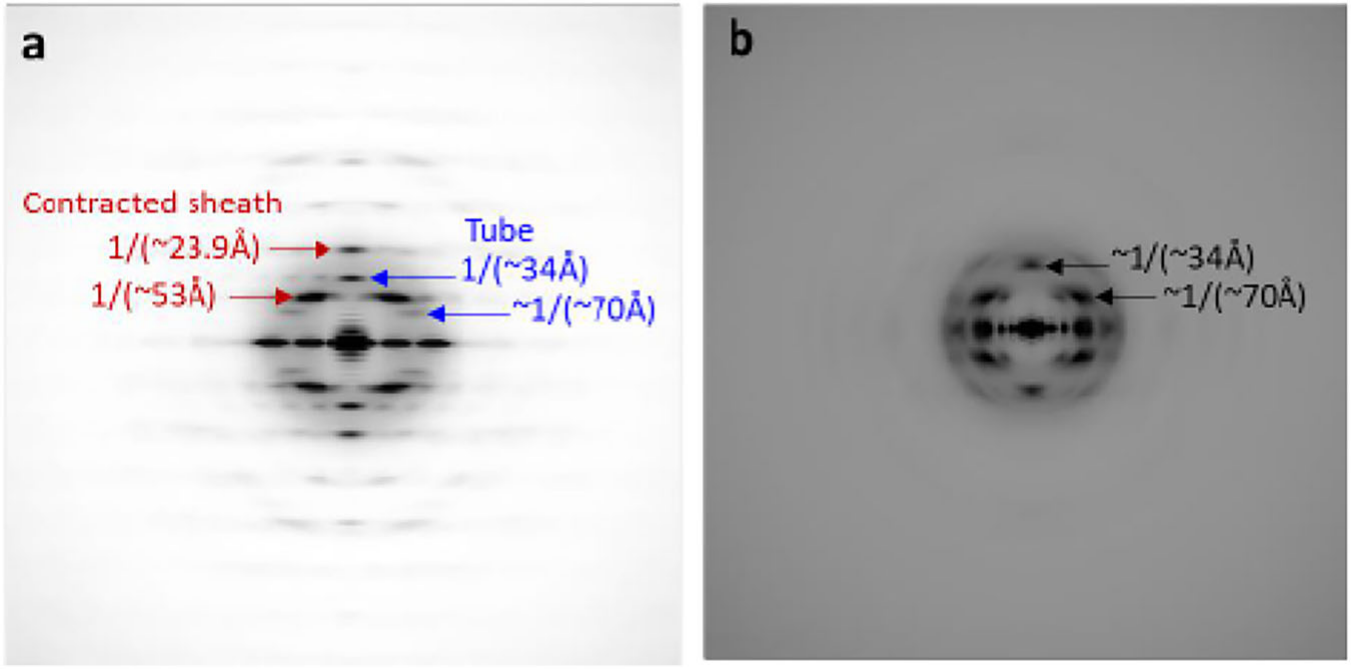


which replaces the previous incorrect version:
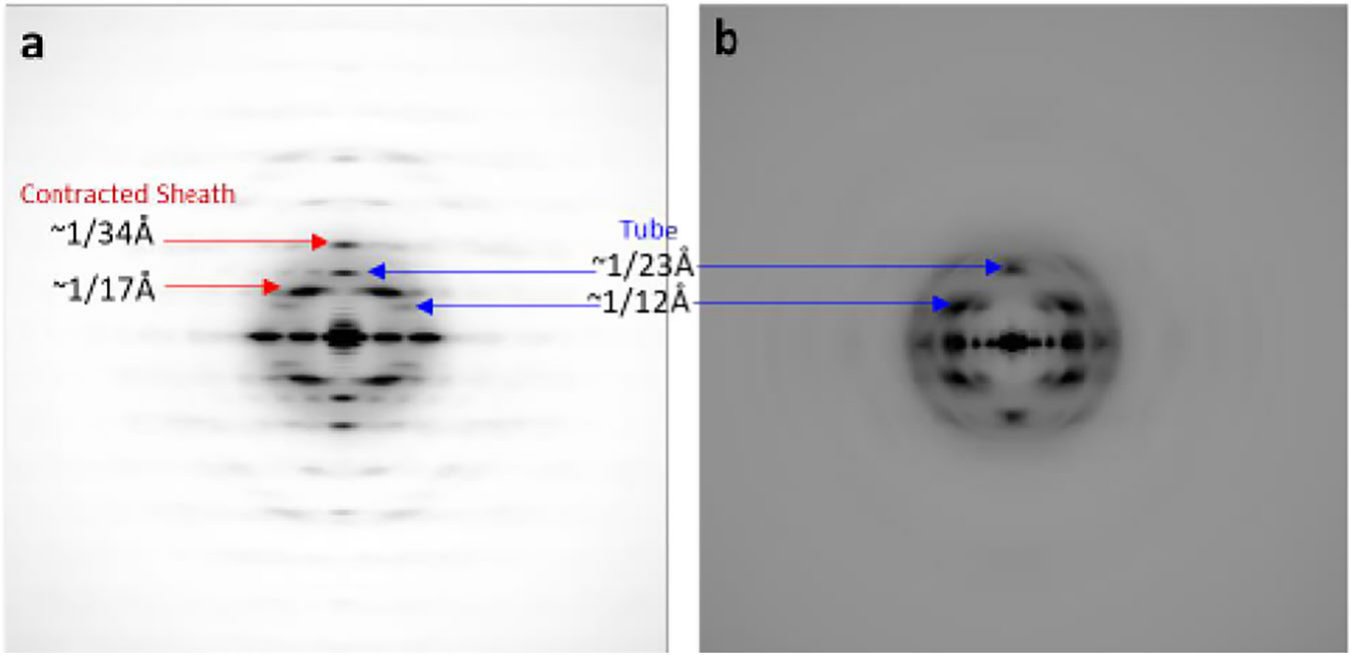


The HTML has been updated to include a corrected version of the Supplementary Information.

## Supplementary information

Revised Supplementary Information

