# Supplementary material for: An extensive disulfide bond network prevents tail contraction in Agrobacterium tumefaciens phage Milano
Source: Nat Commun. 2024 Jan 26;15:756. doi: 10.1038/s41467-024-44959-z (PMC10811340; doi:10.1038/s41467-024-44959-z)
Supplement: Supplementary file 1 — Supplementary Information [file 41467_2024_44959_MOESM1_ESM.pdf]

Supplementary information:

An extensive disulfide bond network prevents tail contraction in *Agrobacterium tumefaciens* phage Milano

Ravi R. Sonani[1], Lee K. Palmer[2], Nathaniel C. Esteves[3], Abigail A. Horton[3], Amanda L. Sebastian[3], Rebecca J. Kelly[3], Fengbin Wang[1,5], Mark A.B. Kreutzberger[1], William K. Russell[2], Petr G. Leiman[4], Birgit E. Scharf[3], Edward H. Egelman[1]

Correspondence to: pgleiman@utmb.edu, bscharf@vt.edu, egelman@virginia.edu

This file contains:
11 Supplementary Figures (S1 – S11) and
Five Supplementary Tables (S1 – S5)

Figure S1

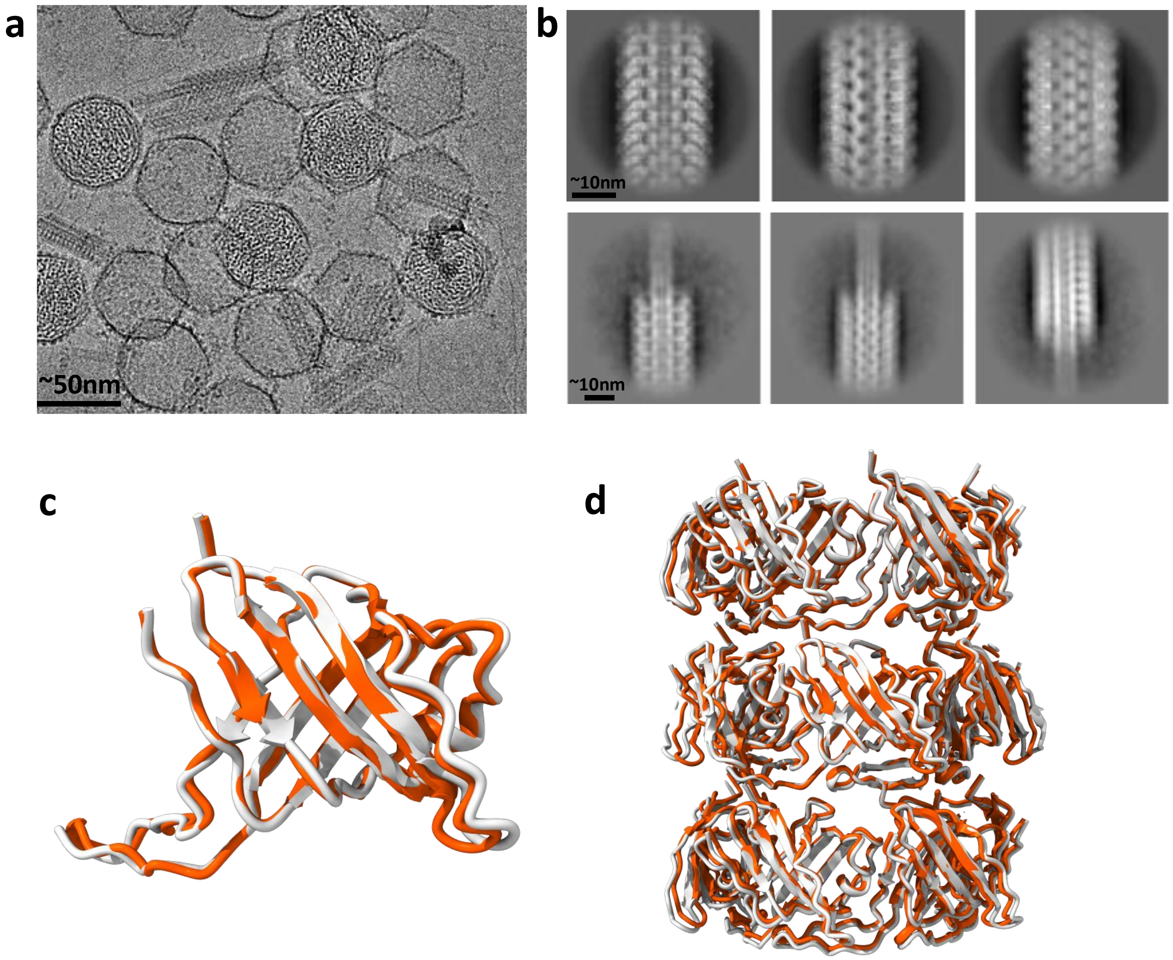

Figure S1: (a) Cryo-EM micrograph of contracted Milano phage. **(b)** Representative 2-D class averages of Milano contracted tail showing its straight morphology. The upper and lower panel show 2D-class averages of middle and end region of the tail. **(c-d)** Superimposed structures of Milano tail-tube (gp21) protomer **(c)** and tube **(d)** in extended (grey) and contracted (orange) state.

Figure S2

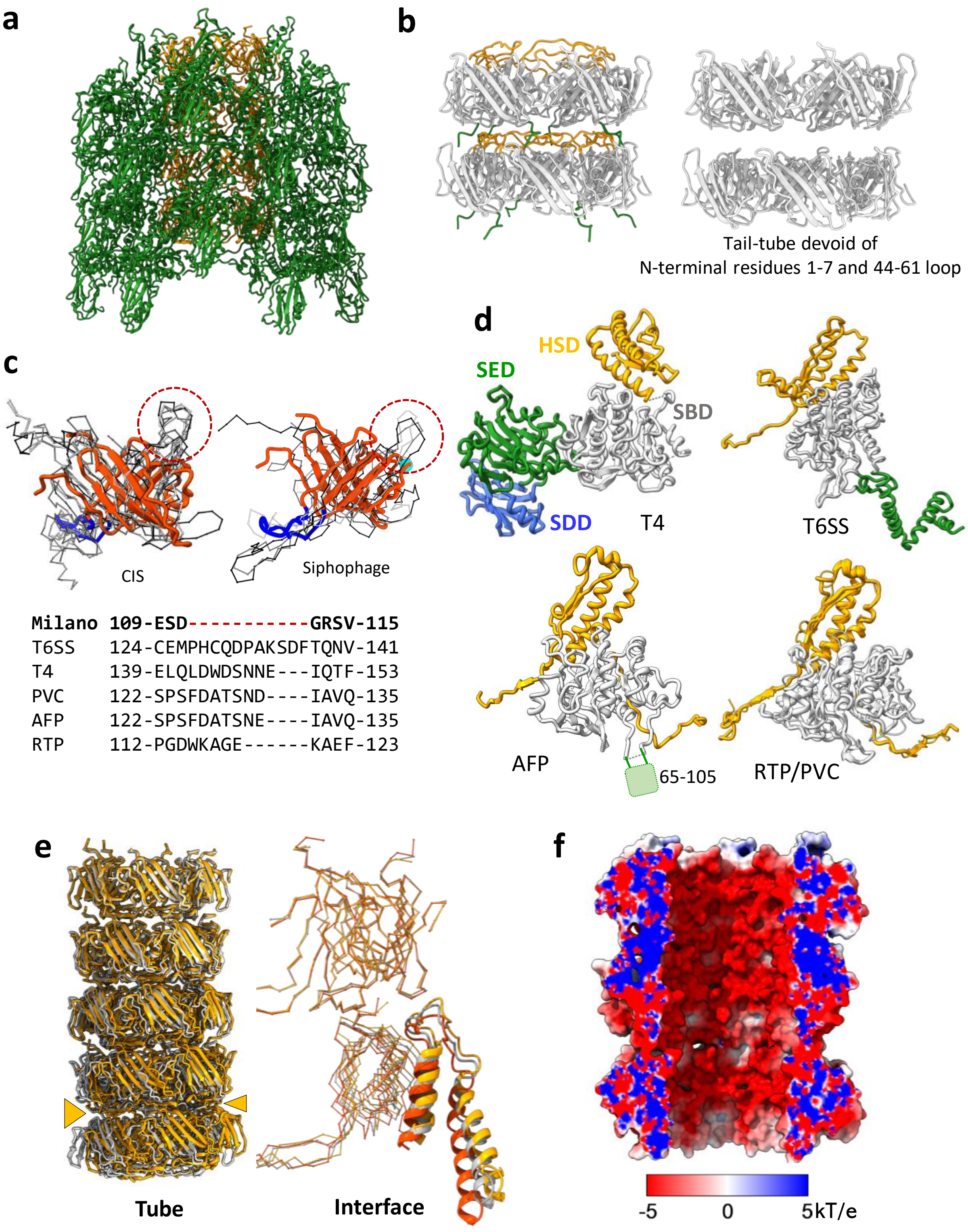

Figure S2: **(a)** Milano tail structure in straight state. Tube (gp21) and sheath (gp20) are colored orange and green, respectively. **(b)** Helical arrangement of gp21 in tube. The yellow-colored

loop (residues 44-61) establishes the contact with neighboring subunit within hexamer. The inter-hexamer contact is mainly mediated by the N-terminal end (residues 1-7) (green) and 44-61 loop (yellow) as removal of which results in no contact between hexamers (Right panel). **(c)** The structure Milano tube protein gp21 superimposed with tube proteins of other CIS (Left panel; T6SS, Type-VI secretion system; T4, T4 bacteriophage; PVC, *Photorhabdus* virulence cassette; AFP, anti-feeding phage; RTP, R-type pyocin) and non-contractile phage tail-tube proteins (Right panel; Phage SPP1, Phage 80α, Phage YSD1). The outer domains of SPP1, 80α and YSD1 are removed for ease of visualization. The sequence alignment (lower panel) reveals absence of specific loop in Milano gp21 (between residues 111-112) compared to tube protein of other CIS. The missing loop is shown by a dashed red circle in the upper structural panel. The lack of this loop results in grooves on Milano tube surface as Shown in main Fig. 1e. **(d)** Structures of sheath proteins of other CISs, T4, T6SS, AFP, RTP and PVC for comparison with Milano sheath protein shown in main Fig. 1f. The sheath handshake (SHD), sheath-body (SBD), sheath-extension (SED), and sheath-decoration (SDD) domains are colored orange, white, green, and blue respectively. (**e**) Comparison of tube and sheath-tube interface between curved (yellow, orange) and baseplate-proximal straight tail segment (grey). Yellow and orange represent subunits at inner and outer surface, respectively. **(f)** Highly negative electrostatic potential of inner surface of Milano tail-tube.

Figure S3

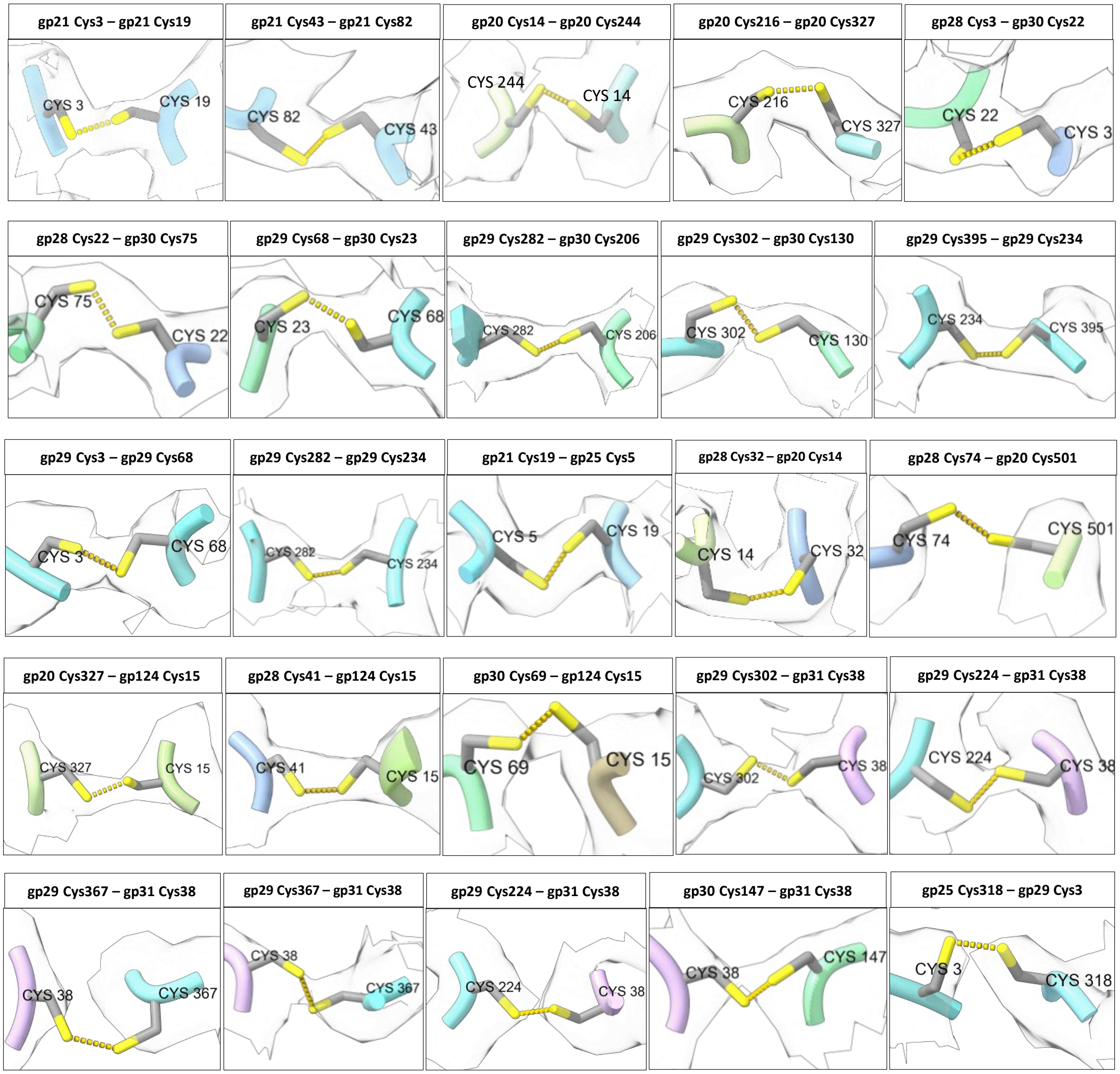

Figure S3: Close up view of representative disulfide bonds in Milano tail and baseplate structure with atomic models shown along with the density map. It can be seen that the density for these bonds is quite variable, with some putative disulfides showing no density for the bond. We note that disulfide bonds are remarkably sensitive to radiation, either x-ray or electrons. It has previously been shown at 2.5 Å resolution that the sensitivity of the disulfide bond to radiation is dependent on the local environment, so that with the same radiation dose the density for some disulfide bonds is almost totally lost while others are retained[1].

Figure S4

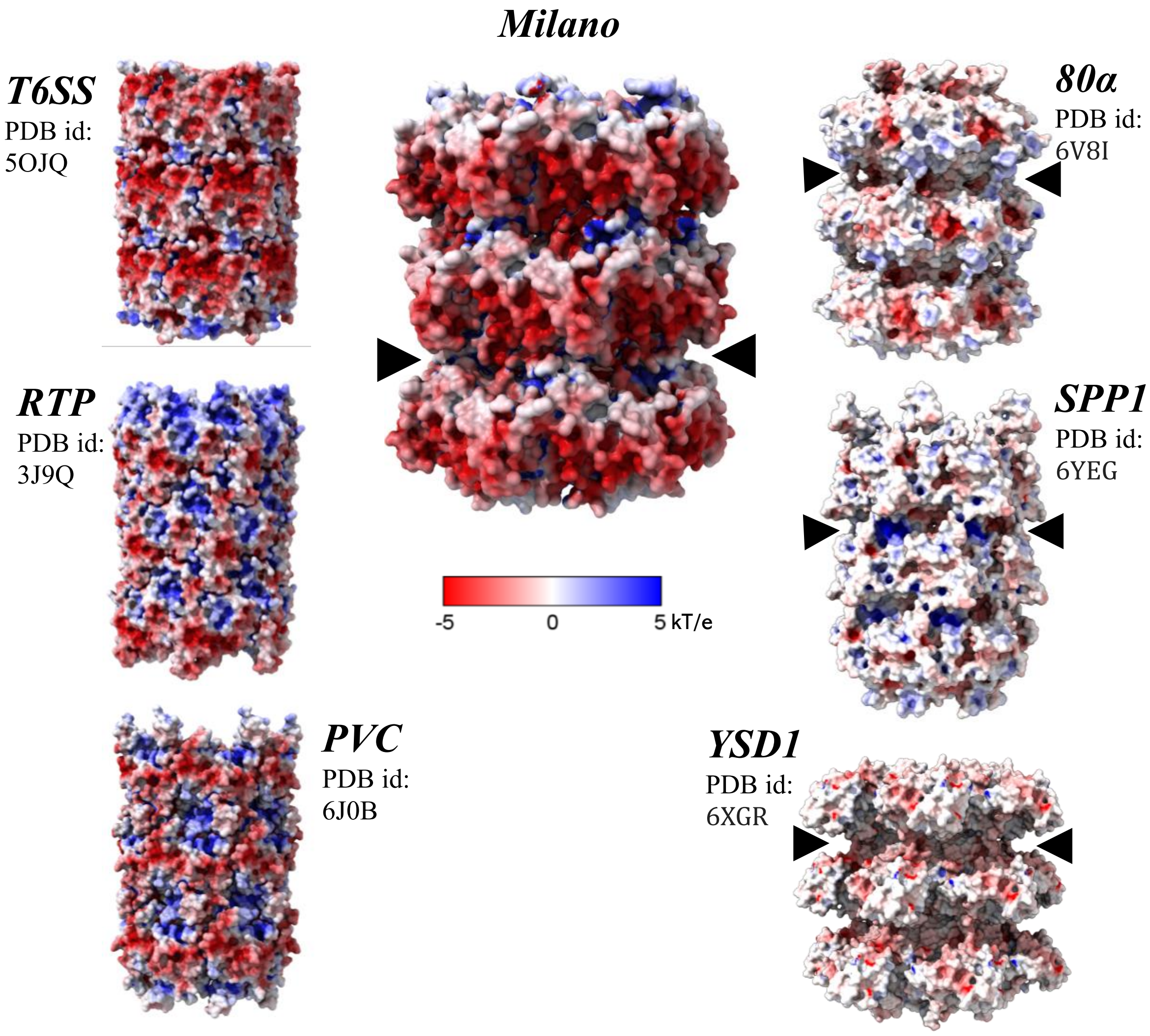

Figure S4: Electrostatic potential and surface morphology of Milano tail tube (gp21) in comparison with other contractile injection system (CIS) (left panel) and tube of Siphophages (right panel). T6SS, Type-VI secretion system; RTP, R-type pyocin; PVC, *Photorhabdus* virulence cassette; 80α, *S. aureus* bacteriophage 80α; SPP1, *Bacillus subtilis* bacteriophage SPP1; *YSD1, Salmonella Typhi* bacteriophage YSD1. Unlike other CIS, Milano tail-tube shows substantial grooves as indicated by arrow heads on its surface similar to Siphophage.

Figure S5

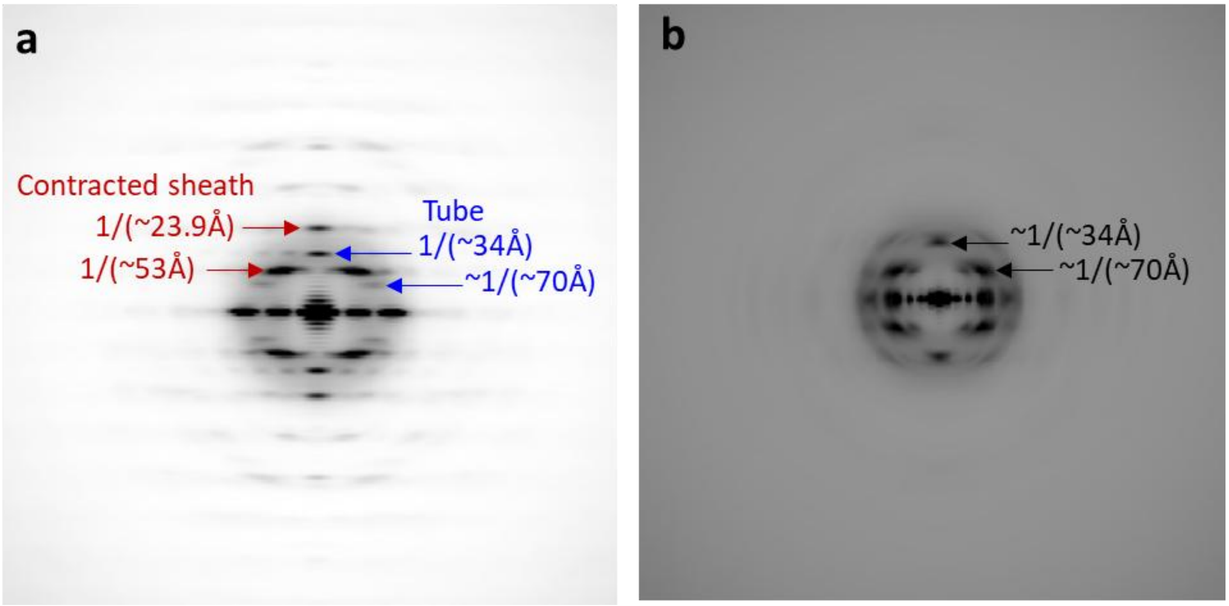

Figure S5: Averaged power spectrum of vertically aligned segments of **(a)** contracted and **(b)** extended tail of Milano. Contracted tail segments were windowed from both sides to include tube and sheath overlapping to it, which decreases the signal from contracted sheath. The additional layer lines from tube-signal appears and allows indexing of tube helical symmetry, which is similar to extended tube symmetry. Layer lines associated with tube and contracted sheath are marked by blue and red arrows, respectively.

Figure S6

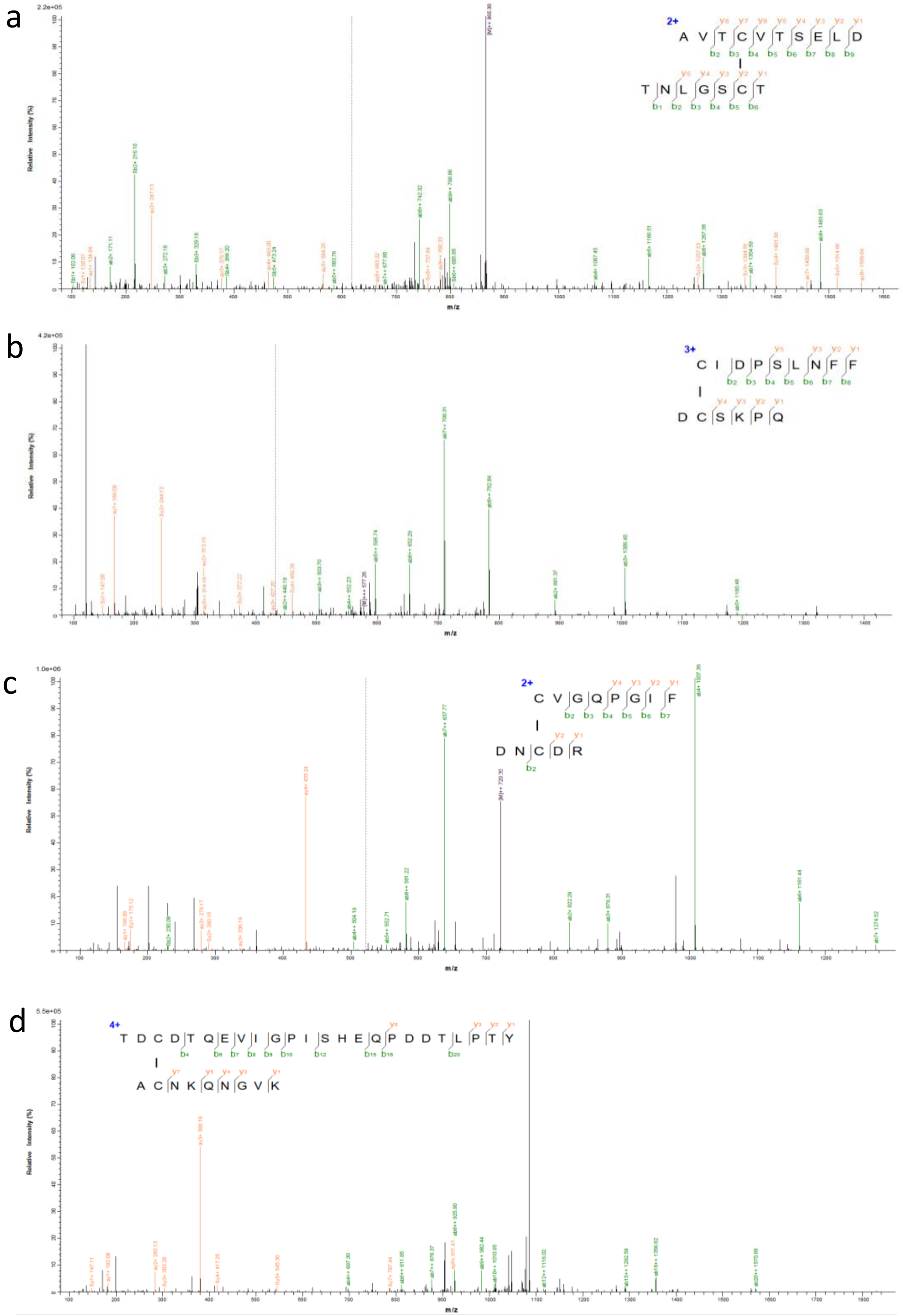

Figure S6: Mass spectrometry fragmentation pattern of the inter-chain disulfide bonds of Milano tail-sheath (gp20) and tail-tube (gp21) showing a peak for doubly charged parent mass. **(a)** gp20 Cys47$_{\text{SBD}}$ - gp20 Cys182$_{\text{SED}}$; **(b)** gp20 Cys14$_{\text{SHD}}$ - gp20 Cys244$_{\text{SBD}}$; **(c)** gp20 Cys467$_{\text{SHD}}$ - gp20 Cys501$_{\text{SHD}}$ and **(d)** gp21 Cys3 - gp21 Cys19

Figure S7

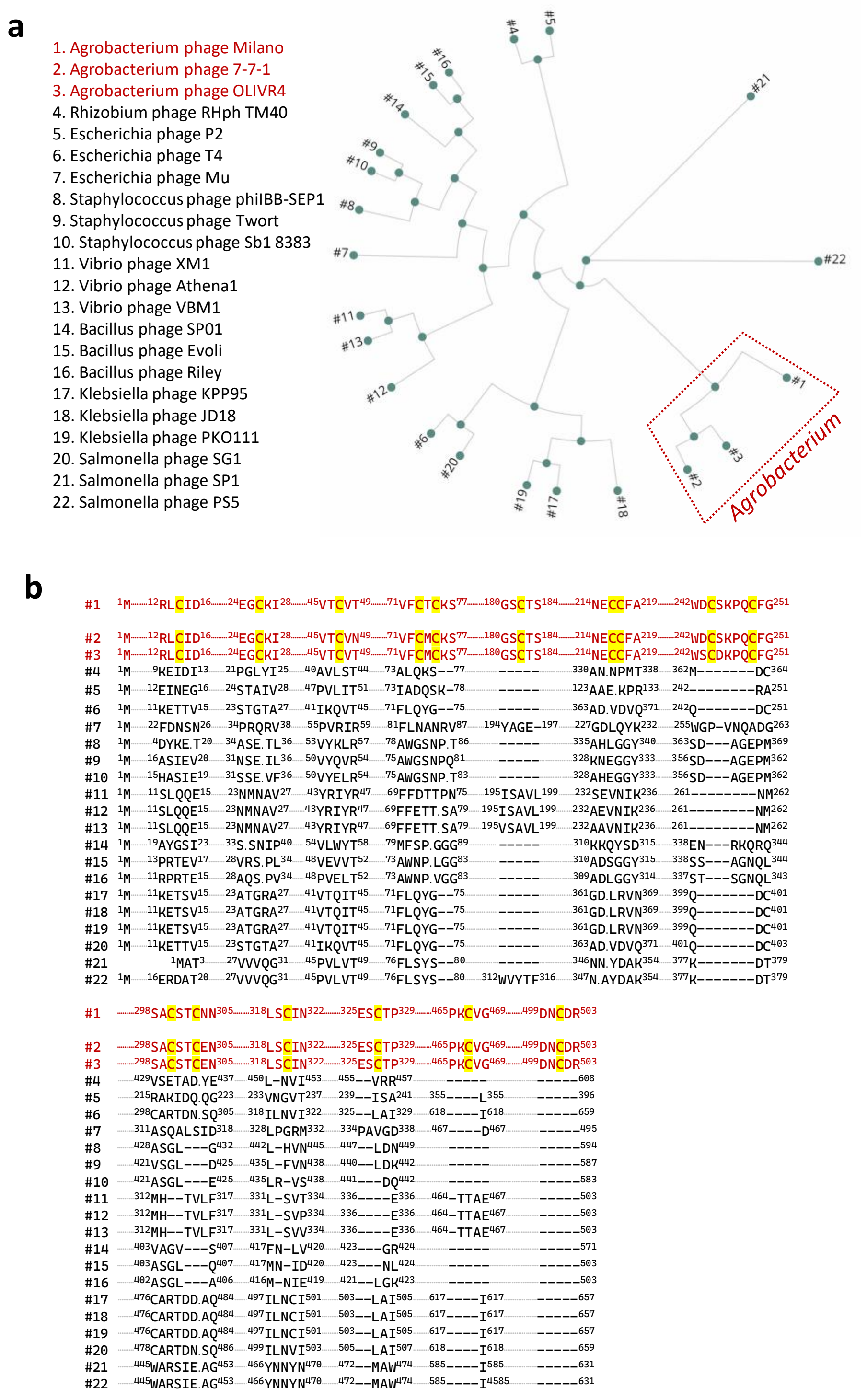

Figure S7: **(a)** Phylogenetic tree of bacteriophages constructed using their sheath protein sequence. **(b)** Structural alignment of Milano sheath protein with other bacteriophage sheath proteins by DALI. Structures were predicted using AlphaFold. The presence of cysteine residues involved in disulfide bonds are conserved in the specific bacteriophage clade infecting the *Agrobacterium*.

Figure S8

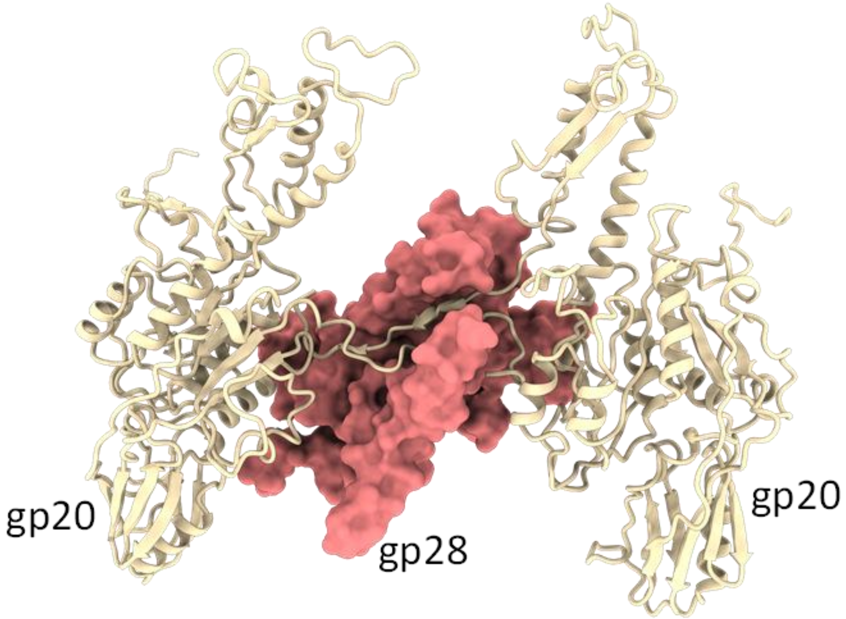

Figure S8: The β-sheet augmentation interaction between baseplate wedge 1 (BW1, gp28) and tail-sheath (gp20) proteins in Milano.

Figure S9

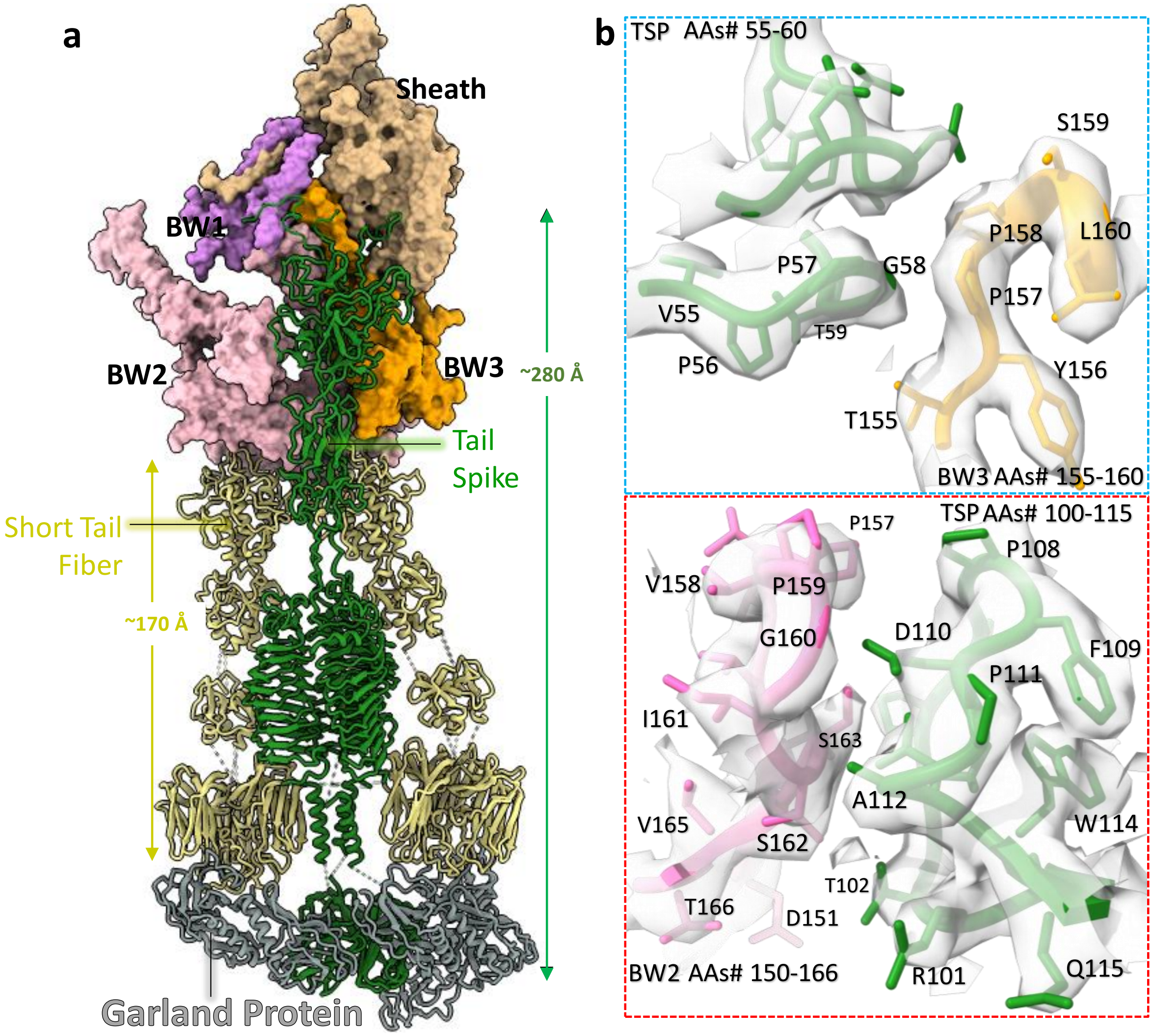

Figure S9: Tail-spike assembly and its interactions with baseplate. (**a**) Tail spike assembly (ribbon model) attached to the baseplate (surface model). Garland protein envelopes the distal parts (C-terminal domains) of TSP and STF proteins. The color scheme is the same as Fig. 3. **(b)** TSP interactions with baseplate wedge proteins: Enlarged view of interacting surfaces (blue and red boxes in Fig. 5b) in ribbon and stick model.

Figure S10

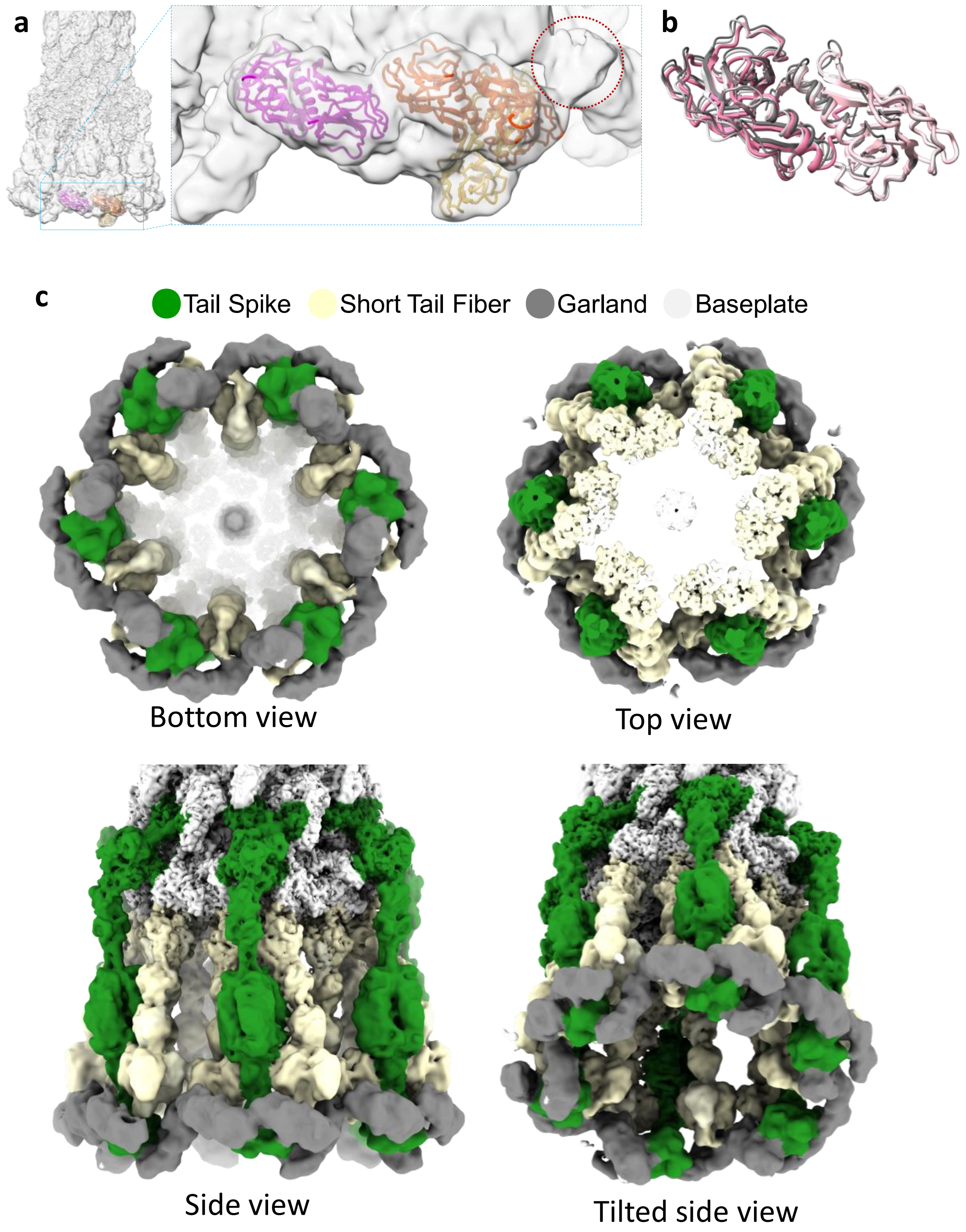

Figure S10: **(a)** Three dimer of garland proteins (gp125$_2$, gp127$_2$ or gp125-gp127) fitted in to cryo-EM map. The red circle indicates the undefined density blob as described in text. **(b)** Superimposed dimer of garland proteins. gp127$_2$, silver; gp125$_2$, grey; gp125-gp127, gp125 and gp127 are colored in dark and light pink, respectively. **(c)** Visualization of the receptor binding complex and garland of Milano depicted through a density map from various perspectives.

Figure S11

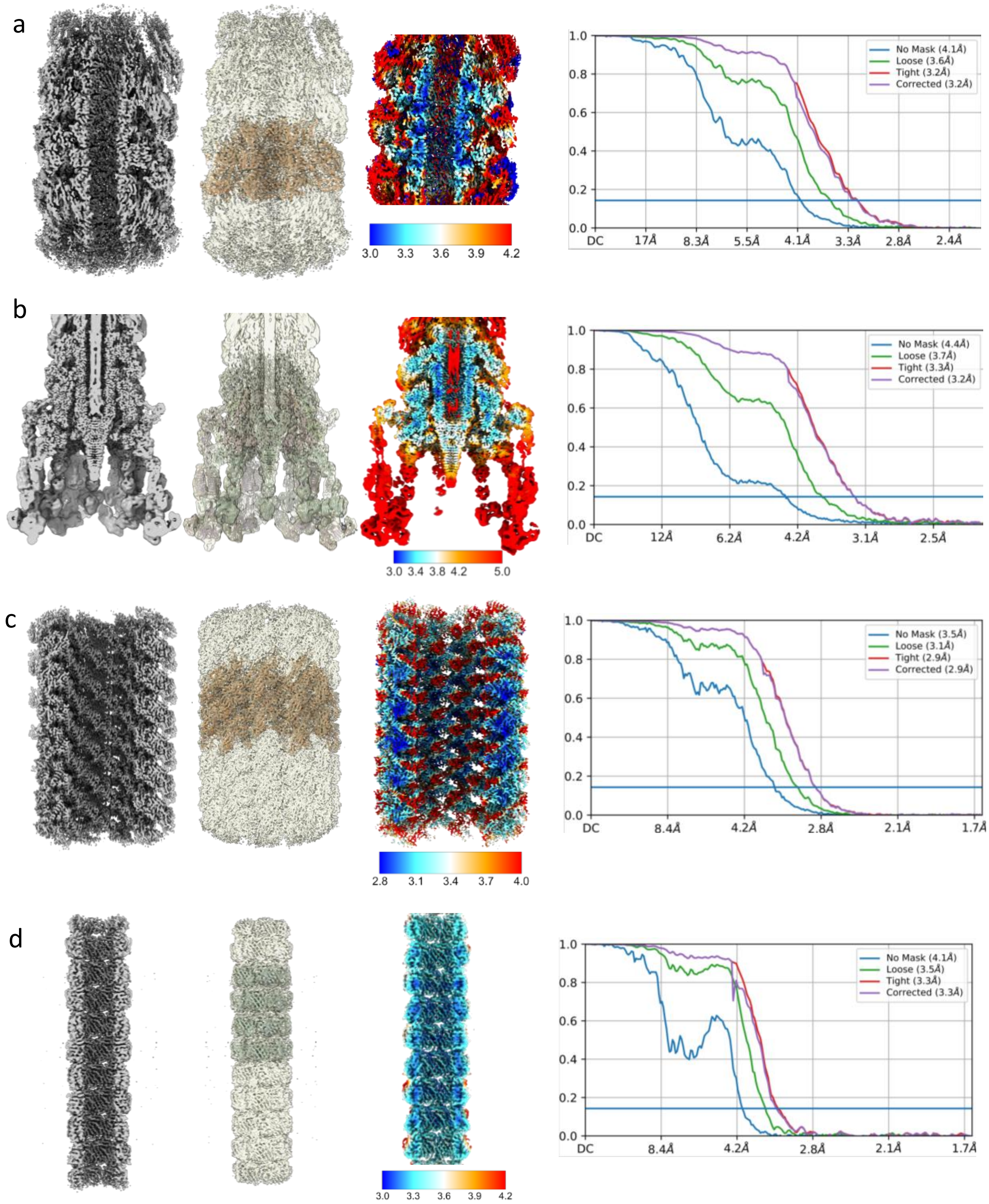

Figure S11: Left-to-right, the experimental density map, model-to-map fitting, local resolution map and map:map FSC calculation curves (with 0.143 cutoff) of all reconstructions described in the manuscript. **(a)** The curved-tail asymmetric reconstruction, **(b)** The C3-symmetrically averaged baseplate reconstruction, **(c)** The contracted tail-sheath helical reconstruction, and **(d)** The contracted tail-tube helical reconstructions, as detailed in Table S5.

Table S1: Identification of the disulfide bonds in Mass Spectrometry data using pLink2[2] (Version 2.3.9, pFind Team, Beijing, China). The E-value is the expectation value for the whole disulfide bonded peptide pair. The Score is the measurement for false discovery rate (FDR) calculation.

Milano disulfides identified	Number of unique peptides representing site	Best scoring peptide sequence	Best E-value	Best Score
gp20 cys 47 - gp20 cys 182	7	AVT**C**VTSEL-TNLGS**C**T	1.95E-12	2.80E-07
gp20 cys 14 - gp20 cys 244	3	**C**IDPSLNFF-D**C**SKPQ	3.31E-14	3.47E-02
gp20 cys 467 - gp20 cys 501	4	**C**VGQPGIF-DN**C**DR	5.10E-11	3.31E-01
gp21 cys 3 - gp21 cys 19	3	A**C**NKQNGVK-TD**C**DTQEVIGPISHEQPDDTLPTY	4.41E-20	1.44E-01

Table S2: Results of DALI structural similarities search for C-terminal domain (residues 170-300) of short tail fiber – gp31

# No	PDB-ChainID	Z	rmsd	lali	nres	%id	Molecules Description
1	6wpx-B	7.9	2.5	89	451	6	BLEST2
2	3afg-B	7.7	2.2	84	507	8	SUBTILISIN-LIKE SERINE PROTEASE
3	5gu5-A	7.1	3.0	86	108	8	TRANSMEMBRANE EMP24 DOMAIN-CONTAINING PROTEIN 5
4	2k46-A	6.5	3.1	92	190	8	MGC80075 PROTEIN
5	2yyo-A	6.5	2.6	97	160	4	SPRY DOMAIN-CONTAINING PROTEIN 3
6	7b2s-A	6.4	3.1	106	172	3	E3 UBIQUITIN-PROTEIN LIGASE TRIM9
7	8beq-A	6.3	3.6	102	534	12	FRUCTOFURANOSIDASE FROM RHODOTORULA DAIRENENSIS
8	6i56-A	6.1	2.9	86	279	10	PHAGE-LIKE ELEMENT PBSX PROTEIN XEPA
9	5azx-A	6.0	2.4	78	100	9	TRANSMEMBRANE EMP24 DOMAIN-CONTAINING PROTEIN 10
10	6nob-A	6.0	3.3	96	634	7	BETA-FRUCTOFURANOSIDASE
11	3ldk-A	5.9	3.4	109	634	11	FRUCTOSYLTRANSFERASE
12	2qfe-A	5.9	2.4	84	139	6	CALPAIN-7
13	2wfk-A	5.9	3.1	102	255	3	LIPL32
14	7oa8-A	5.8	3.6	104	376	7	PILC MINOR PILIN
15	3o12-A	5.7	3.1	101	185	3	UNCHARACTERIZED PROTEIN YJL217W
16	4uyb-A	5.6	2.6	83	400	6	SEC14-LIKE PROTEIN 3
17	4ffg-A	5.6	3.7	108	480	6	LEVAN FRUCTOTRANSFERASE
18	5iku-A	5.6	2.2	79	240	9	COLLAGENASE
19	7js4-A	5.6	2.9	86	953	7	F5/8 TYPE C DOMAIN PROTEIN
20	7nsb-a	5.5	3.4	106	568	5	VACUOLAR IMPORT AND DEGRADATION PROTEIN 30
21	8bv0-B	5.5	2.9	97	206	9	NRC1
22	6e57-A	5.5	2.6	86	399	8	SURFACE GLYCAN BINDING PROTEIN B
23	6s44-A	5.4	3.3	90	146	6	CAPSID PROTEIN
24	6r3u-A	5.4	3.3	106	495	4	GLYCOSIDE HYDROLASE FAMILY 32
25	2bvb-A	5.3	3.2	89	137	8	MICRONEMAL PROTEIN 1

Table S3: Identification of structural proteins in Milano by mass spectrometry. Procedure: The Milano phage sample was reduced and alkylated with carbamidomethylated before digestion by trypsin. Digested phage sample in 0.1% formic acid is injected on a Dionex Ultimate 3000 nano-LC system (Thermo Scientific) coupled to a Bruker Maxis Impact QTOF mass spectrometer for MS/MS analysis. The results were analyzed against the Milano proteome using Mascot software.

<table>
<tr><th>Id#</th><th>Id</th><th>Protein name</th><th>Coverage</th><th>MW</th><th>Score</th></tr>
<tr><td>1.1</td><td>tr|A0A482MFS6|A0A482MFS6_9CAUD</td><td>Major capsid protein OS=Agrobacterium phage Milano OX=2557550 GN=Milano_009 PE=4 SV=1</td><td>60.9 %</td><td>52512</td><td>2163</td></tr>
<tr><td>1.2</td><td>tr|A0A482MHM7|A0A482MHM7_9CAUD</td><td>Tape measure protein OS=Agrobacterium phage Milano OX=2557550 GN=Milano_024 PE=4 SV=1</td><td>43.3 %</td><td>82296</td><td>1333</td></tr>
<tr><td>1.3</td><td>tr|A0A482MFS8|A0A482MFS8_9CAUD</td><td>Tail sheath protein OS=Agrobacterium phage Milano OX=2557550 GN=Milano_020 PE=4 SV=1</td><td>33.6 %</td><td>54739</td><td>940</td></tr>
<tr><td>1.4</td><td>tr|A0A482MFU4|A0A482MFU4_9CAUD</td><td>Baseplate protein OS=Agrobacterium phage Milano OX=2557550 GN=Milano_029 PE=4 SV=1</td><td>47.5 %</td><td>44235</td><td>916</td></tr>
<tr><td>1.5</td><td>tr|A0A482MFW7|A0A482MFW7_9CAUD</td><td>Portal protein OS=Agrobacterium phage Milano OX=2557550 GN=Milano_007 PE=4 SV=1</td><td>32.9 %</td><td>45931</td><td>571</td></tr>
<tr><td>1.6</td><td>tr|A0A482MHC6|A0A482MHC6_9CAUD</td><td>Virion-associated protein OS=Agrobacterium phage Milano OX=2557550 GN=Milano_001 PE=4 SV=1</td><td>18.7 %</td><td>52901</td><td>559</td></tr>
<tr><td>1.7</td><td>tr|A0A482MHF3|A0A482MHF3_9CAUD</td><td>Virion-associated protein OS=Agrobacterium phage Milano OX=2557550 GN=Milano_031 PE=4 SV=1</td><td>32.0 %</td><td>32141</td><td>480</td></tr>
<tr><td>1.8</td><td>tr|A0A482MFQ9|A0A482MFQ9_9CAUD</td><td>DNA-circularization protein OS=Agrobacterium phage Milano OX=2557550 GN=Milano_025 PE=4 SV=1</td><td>26.4 %</td><td>43392</td><td>441</td></tr>
</table>

1.9	tr|A0A482MF64|A0A482MF64_9CAUD	Prohead protease OS=Agrobacterium phage Milano OX=2557550 GN=Milano_008 PE=4 SV=1	24.2 %	28724	400
1.10	tr|A0A482MHL8|A0A482MHL8_9CAUD	Head-to-tail connector complex protein OS=Agrobacterium phage Milano OX=2557550 GN=Milano_014 PE=4 SV=1	44.1 %	22447	341
1.11	tr|A0A482MFR0|A0A482MFR0_9CAUD	Virion-associated protein OS=Agrobacterium phage Milano OX=2557550 GN=Milano_016 PE=4 SV=1	40.6 %	23111	327
1.12	tr|A0A482MFS0|A0A482MFS0_9CAUD	Virion-associated protein OS=Agrobacterium phage Milano OX=2557550 GN=Milano_010 PE=4 SV=1	48.9 %	14761	290
1.13	tr|A0A482MGH3|A0A482MGH3_9CAUD	Virion-associated protein OS=Agrobacterium phage Milano OX=2557550 GN=Milano_013 PE=4 SV=1	16.1 %	30801	286
1.14	tr|A0A482MFT7|A0A482MFT7_9CAUD	Virion-associated protein OS=Agrobacterium phage Milano OX=2557550 GN=Milano_030 PE=4 SV=1	36.0 %	32169	284
1.15	tr|A0A482MGV0|A0A482MGV0_9CAUD	Putative tail fiber protein OS=Agrobacterium phage Milano OX=2557550 GN=Milano_124 PE=4 SV=1	10.4 %	61866	277
1.16	tr|A0A482MGG2|A0A482MGG2_9CAUD	Uncharacterized protein OS=Agrobacterium phage Milano OX=2557550 GN=Milano_003 PE=4 SV=1	22.0 %	48545	230

<table>
<tr><td>1.17</td><td>Unknown_structurally_identified</td><td>nan
gp28</td><td>20.8 %</td><td>19628</td><td>201</td></tr>
<tr><td>1.18</td><td>tr|A0A482MFQ3|A0A482MFQ3_9CAUD</td><td>Head completion protein OS=Agrobacterium phage Milano OX=2557550 GN=Milano_015 PE=4 SV=1</td><td>35.5 %</td><td>16191</td><td>194</td></tr>
<tr><td>1.19</td><td>tr|A0A482MF73|A0A482MF73_9CAUD</td><td>Virion-associated protein OS=Agrobacterium phage Milano OX=2557550 GN=Milano_018 PE=4 SV=1</td><td>23.0 %</td><td>20349</td><td>180</td></tr>
<tr><td>1.20</td><td>tr|A0A482MG23|A0A482MG23_9CAUD</td><td>Virion-associated protein OS=Agrobacterium phage Milano OX=2557550 GN=Milano_127 PE=4 SV=1</td><td>30.8 %</td><td>14183</td><td>179</td></tr>
<tr><td>1.21</td><td>tr|A0A482MFZ0|A0A482MFZ0_9CAUD</td><td>Baseplate protein OS=Agrobacterium phage Milano OX=2557550 GN=Milano_027 PE=4 SV=1</td><td>17.0 %</td><td>20739</td><td>133</td></tr>
<tr><td>1.22</td><td>tr|A0A482MFS1|A0A482MFS1_9CAUD</td><td>Baseplate hub protein OS=Agrobacterium phage Milano OX=2557550 GN=Milano_026 PE=4 SV=1</td><td>8.1 %</td><td>49788</td><td>123</td></tr>
<tr><td>1.23</td><td>tr|A0A482MHX6|A0A482MHX6_9CAUD</td><td>Uncharacterized protein OS=Agrobacterium phage Milano OX=2557550 GN=Milano_125 PE=4 SV=1</td><td>29.8 %</td><td>14536</td><td>93</td></tr>
</table>

Table S4: Intra-chain disulfide bonds in Milano tail and baseplate structure

Sheath (gp20)	Cys26	Cys303
	Cys73	Cys320
	Cys217	Cys249
	Cys75	Cys300
Baseplate wedge 2 (gp29)	Cys69	Cys181
	Cys173	Cys212
	Cys223	Cys374
	Cys250	Cys379
Baseplate wedge 3 (gp30)	Cys33	Cys66
	Cys125	Cys264
	Cys212	Cys277
Short tail fiber (gp31)	Cys12	Cys20

Table S5: Cryo-EM data collection and refinement statistics of Milano tail and baseplate junction structures

	**Curved tail**	**Baseplate**	**Contracted tube**	**Contracted sheath**
PDB entry	8FOP	8FQC	8FOU	8FOY
EMBD entry	29353	29383	29354	29355
Voltage (kV)	300	300	300	300
Magnification (x)	81,000	81,000	105,000	105,000
Electron exposure (e^{-}/ $Å^2$)	50	50	48	48
Pixel size (Å/pixel)	1.08	1.08	0.82	0.82
Particle images (no.)	228,393	14,856	160,399	160,399
Box size (Å)	384	578	512	512
**Symmetry imposed**				
	Point group: C1	Point group: C3	Helical<br>Rise: 34.068 Å<br>Twist: 28.164˚<br>Point group: C6	Helical<br>Rise: 23.934 Å<br>Twist: -30.562˚<br>Point group: C6
**Map global resolution (Å)**				
Map:map Fourier shell correlation (FSC, 0.143)	3.2 Å	3.2 Å	3.3 Å	2.9 Å
Model:map FSC (0.5)	3.5 Å	3.4 Å	3.5 Å	2.9 Å
**Refinement and model validation**				
Clash score	9.37	10.20	8.88	9.08
R.M.D deviations				
Bond Length (Å)	0.003	0.007	0.003	0.009
Bond Angle (°)	0.563	0.793	0.602	0.783
Ramachandran plot (%)				
Outliers	0.57	0.75	0.00	0.20
Allowed	7.14	8.75	4.80	9.04
Favoured	92.29	90.49	95.20	90.76
Model vs. Data fit				
CC (mask)	0.80	0.85	0.85	0.90
CC (Volume)	0.79	0.84	0.83	0.89

References

1. Pieri, L. et al. Atomic structure of Lanreotide nanotubes revealed by cryo-EM. *Proc Natl Acad Sci U S A* **119**(2022).
2. Chen, Z.-L. et al. A high-speed search engine pLink 2 with systematic evaluation for proteome-scale identification of cross-linked peptides. *Nature communications* **10**, 3404 (2019).
