# Supplementary material for: An extensive disulfide bond network prevents tail contraction in Agrobacterium tumefaciens phage Milano
Source: Nat Commun. 2024 Jan 26;15:756. doi: 10.1038/s41467-024-44959-z (PMC10811340; doi:10.1038/s41467-024-44959-z)
Supplement: Supplementary file 3 — Description of Additional Supplementary Files [file 41467_2024_44959_MOESM3_ESM.docx]

**Description of additional supplementary information files**

**Title:** Supplementary Movie 1

**Description:** Morphing of the Milano sheath between the extended and contracted states. The models of the extended and contracted sheath assemblies were aligned by the top hexametric ring and morphed in ChimeraX. The non-physiological extension process is shown for continuity. Cysteines involved in disulfide bonds are shown by cyan ((Cys14_SHD_:Cys244_SBD_), magenta (Cys216_SBD_:Cys327_SBD_) and yellow colored (Cys47_SBD_:Cys182_SED_) spheres. During wave-like sheath contraction, the distance between Cys47_SBD_ and Cys182_SED_ (cyan colored) increases to a greater degree than in the synchronous motion shown here. The disulfide bond Cys47_SBD_:Cys182_SED_ is broken in the contracted sheath as inter-Cα distances between Cysteines is increased to ~7.5 Å and their Sγ atoms are separated by 3.0 Å with a density blob that might correspond to a DTT molecule.

**Title:** Supplementary Movie 2

**Description**: Same as Supplementary Movie 1, but a top view

**Title:** Supplementary data 1

**Description:** The PDB file of the structure of baseplate with full receptor binding proteins. This model is generated by combining the baseplate structure (PDB id: 8FQC) and rigid body fitted AlphaFold models of TSP (residues 175-587), STF (residues 125-300) and garland proteins.
